# Consumption

**Published:** 1860-03

**Authors:** J. G. Westmoreland


### ARTICLE III.

*Consumption.*—By J. G. WESTMORELAND, M. D. [Continued from page 274.]

A peculiar constitutional formation seems indispensable to the existence of true tubercular phthisis. This is called the strumous or tubercular diathesis, in contradistinction to other constitutional impresses, disposing to the development of other abnormal conditions.

This peculiarity is not disease, nor does it necessarily induce unhealthy action, but requires that the system be brought into the proper condition for its development before that occurs. This peculiar conformation is either inherited, and exists from birth, or is contracted, and probably consists in the molecular arrangement differing from what is usual, so as to modify the special irritability of the tissue involved. A physician of this city, Dr. Coe, whose opinions are generally sustained by sound reasoning, contends for the special irritability of every tissue, and that it may be so modified that its specific stimulus may cease to excite the proper degree of action, or may super-excite, according to the change produced. Be that, however, as it may, there is doubtless a constitutional liability to particular diseases, to be developed by certain direct causes. The rheumatic predisposition, or diathesis, exists in certain individuals, and rheumatism may be developed in such by exposure to dampness or cold, or whatever is likely to excite an attack, even in one of plethoric habit and robust health. The consumptive or tuberculous subject has a like persistent predisposition to the development of another character of disease, and requires a different state of things for its attack.

While rheumatism, gout, &c., may attack the nervous and strong, phthisis and scrofula are developed only in the impoverished and enfeebled system, and the object in view, in calling attention to the subject under consideration, is to impress some, as I conceive, important practical considerations in the management of tuberculous patients, viz: the necessity of correct diagnosis and the promotion, by every available means, of the nourishment and strength of the body. Many having the tuberculous diathesis, no doubt, perish, who by conforming to the proper course of living, might be spared to their families and friends without medicine, and even without the development of the disease at all.

The greatest difficulty in the management of phthisis pulmonalis, aside from the intractable nature of the disease, is the very general prevalence of the opinion that consumption is incurable. Now, faith has, perhaps, no direct influence in the operation of plans of treatment, at all times, but he who has worried with a despairing patient, in order to have his directions carried out, can very well understand the powerfully indirect influence exerted sometimes by confidence.

The danger of this so generally received opinion is not confined to those actually affected with this disease; for, inasmuch as consumptives have cough, as one of the general prominent symptoms, when it is somewhat persistent from any character of derangement of the respiratory organs, those who make out their diagnosis in a large degree by guess, and, at the same time, are not very particular whether they guess right or wrong, are very likely to pronounce a simple irritation of the larynx or epiglotis "confirmed consumption," and, at the same time, endorse the opinion of its incurability. Such cases are subject to speedy relief, which, if suffered to take their course, may result in troublesome chronic bronchitis.

[To be Continued.]

